# Blood and Blood: Anti-retroviral Therapy, Masculinity, and Redemption among Adolescent Boys in the Eastern Cape Province of South Africa

**DOI:** 10.1111/maq.12686

**Published:** 2022-01-14

**Authors:** Lesley Gittings, Christopher J. Colvin, Rebecca Hodes

**Affiliations:** Centre for Social Science Research, University of Cape Town and Factor-Inwentash Faculty of Social Work University of Toronto; Department of Public Health Sciences, University of Virginia, Division of Social and Behavioural Sciences School of Public Health and Family Medicine, University of Cape Town, and Department of Epidemiology, Brown University; Centre for Sexualities, AIDS & Gender, University of Pretoria and Department of Historical and Heritage Studies University of Pretoria

**Keywords:** masculinity, South Africa, HIV, adolescence

## Abstract

Adolescents living with perinatally acquired HIV are among the first generation in South Africa to grow up with anti-retroviral therapy and democratic freedoms. In this article, we explore the biosocial lives of adolescent boys and young men living with HIV in the Eastern Cape Province of South Africa. We conducted qualitative research with 36 adolescent boys and young men in 2016–2018, including life history narratives, semi-structured interviews, and analysis of health facility files.

## Introduction

In July 2000, Xolani Nkosi Johnson made history when he spoke at the 13th International AIDS Conference in Durban. He was 11 years old, and at the time, the oldest-known child born with HIV. An activist, he called for the South African government to roll out zidovudine to prevent mother-to-child transmission of HIV. He died in 2001 at the age of 12.

Fortunately, things have changed since Nkosi’s death. The HIV treatment landscape in South Africa has been radically transformed. Although there is still no cure, anti-retroviral therapy (ART) is available at public health facilities via the largest public HIV program in the world (Burton et al. 2015). ART provides pregnant mothers the possibility of preventing the transmission of HIV to their infants, and, for children born with HIV, the possibility to live healthily into adulthood. However, health and social systems continue to fail many adolescents living with HIV. Many have persistent, dismal health outcomes due to challenges with ART adherence and retention in care (Hudelson and Cluver 2015).

The HIV epidemic is gendered. Women and girls are more likely to contract HIV for biological and social reasons, and men are more likely to die of AIDS-related illness, less likely to adhere to ART, and less likely to be retained in HIV care (Cornell et al. 2011). The need to better engage men and boys in HIV prevention and treatment initiatives is increasingly acknowledged (Colvin 2019). However, Mfecane (2018) suggests that gender and health interventions in Southern Africa have seen limited success because they are not adequately grounded in local contexts and theories.

Evidence on the biosocial lives of adolescent boys is needed. Despite poor HIV outcomes amongst adolescents and men, masculinity-related aspects of HIV-positive adolescence remain understudied, although older adolescent boys may have poorer ART adherence and viral suppression than their female counterparts (Brittain et al. 2017).

This article explores two powerful organizing factors in the lives of adolescent boys living with HIV in South Africa’s Eastern Cape Province—masculinity and an HIV-positive status. It considers how participants navigate these aspects of their lives, alongside intersecting identities as young Xhosa people, growing up in contexts of precarity and constraint.

## Methods

This research was a sub-study of a longitudinal, mixed-methods study of medicines-taking of adolescents living with HIV. It focused on the health practices of adolescent boys living with HIV (*n* = 35, ages 13–22). Data were collected in rural, urban, and peri-urban areas in the Buffalo City Metropolitan Municipality and Amathole District of the Eastern Cape Province of South Africa in 2016–2018. Adolescent participants identified as male, were living with perinatally acquired HIV, had initiated onto ART, spoke isiXhosa, and grew up within isiXhosa-speaking households.

Art-based life history narrative interviews (*n* = 35) were the primary source of data (participant details are provided in Appendix 1). Life history narratives involve narrating life experiences and highlighting aspects in a domain of inquiry. Participants were asked to share about their lives, including a minimum of three important events. Following this, they were asked to overlay health events onto their life histories. Participants could draw, write, or speak about life events. The metaphor of a “river of life,” was offered as an art-based tool to allow for alternatives to verbal expression using paper and writing and coloring materials. They could also select and trace items such as stones and glass of different colors and shapes to represent themselves and members of their family. Emoji stickers offered the option to represent feelings using social media imagery.

In-depth, semi-structured interviews (*n* = 32) were conducted one to four months following the initial interview and elicited information about perspectives on masculinity and biomedical and traditional health-seeking. For those who attended *ulwaluko* (traditional initiation/circumcision) during the study period (*n* = 5), additional interviews were conducted before and after. Health facility files (*n* = 41 files, 30 participants) were photographed, and analyzed alongside interview data. Interviews were conducted by two young, isiXhosa-speaking male researchers. The lead author supervised this research but did not sit in on most adolescent interviews, given the sensitivities of discussing amaXhosa male rites of passage. Immediately following each interview, the lead author and researcher who conducted the interview discussed each interview in detail, taking notes. Interviews were audio-recorded, translated into English, and transcribed. Visual outputs were photographed. In-depth semi-structured interviews were conducted with traditional and biomedical health workers (*n* = 14) by the lead author. We engaged Braun and Clarke’s (2006) approach to thematic analysis. Nvivo software was used to assist in data management and analysis.

Ethics approvals were provided by the University of Cape Town (HREC 314/2017) and the Eastern Cape Department of Health (EC_201709_13). Informed consent was provided by all participants, and in the case of legal minors, their caregivers.

### Study Setting

This research was conducted in former Eastern Cape “homelands,” designated for Black residents during apartheid, and deliberately underdeveloped and under-resourced. Labor laws forced Black men to leave their families behind to travel to work in mines and urban areas, intentionally weakening family structures (Nell et al. 1997). Today, the Eastern Cape is one of the country’s poorest provinces, with the lowest access to water and electricity in South Africa and the highest proportion of households that had skipped a meal in the past year (Statistics South Africa 2016). The HIV prevalence among adults (ages 15–49) is 25.2%, higher than South Africa’s national prevalence of 20.6% (Statistics South Africa 2016).

### HIV in South Africa

The HIV epidemic presented a grave and devastating contrast to the celebration and hope of South Africa’s transition to democracy (Hodes 2014). HIV emerged in the 1980s, and rates increased significantly in the 1990s. When HIV prevention and treatment medicines became available, President Thabo Mbeki and Minister of Health Mantombazana Tshabala-Msimang questioned the causal link between HIV and AIDS and the efficacy and safety of ART. They refused to make ART available until forced to do so by a constitutional court ruling in 2002 (Heywood 2003). This delayed implementation resulted an estimated 330,000 deaths, and the widespread and avoidable transmission of HIV (Burton et al. 2015), including 35,000 babies born with HIV (Chigwedere et al. 2008). The roll-out of PMTCT and ART was then slow and uneven across the country, and the Eastern Cape in particular lagged behind the rest of the country (Burton et al. 2015). This research was conducted in the Eastern Cape with adolescent boys born with HIV between 1995 and 2004. Many are among the millions of children born in this period who became orphaned (Burton et al. 2015).

## Definitions and Conceptual Frameworks

### Personhood and Redemption

The concept of personhood is employed as a framework in this article,^1^ with three relevant aspects (Mfecane 2018). First, humans have physical and nonphysical elements (De Craemer 1983; Mbiti 1990; Menkiti 2004; Mfecane 2018). Second, personhood is relational—not only in the sense of the living, but kinship relationships with those who have passed on (De Craemer 1983; Mbiti 1990; Menkiti 2004; Mfecane 2018). For this reason, kinship relationships are central to HIV research and programming, where cultural and biological understandings of HIV are deeply intertwined (Block and McGrath 2019). Third, personhood is acquired progressively throughout life, including through rituals of incorporation (Menkiti 1979, 2004). Rituals of incorporation within the study site include *imbeleko*, which serves to introduce one to the ancestors, and ulwaluko, which serves to legitimize them as adult men. Notions of personhood within this study site also have gendered aspects. Ulwaluko defines manhood socially, as well as physically (Mfecane 2020). The process of becoming respectful and respectable men is also grounded in relationships with families and communities, and other gendered pathways to personhood include having children who will carry on their names and heading a household (Gibbs et al. 2014). This research considers these contextually grounded notions of personhood, which are an often-missing feature of research and practice with men in South Africa (Mfecane 2018).

Ancestral beliefs and traditional^2^ cosmologies of health are central to this research. They encompass not just the physical body, but also relationships, family systems, community, place, and history (De Craemer 1983; Mfecane 2018). Familial, personal, and societal misfortunes are often discussed as related to wider social ills rather than biological disease (Becker and Geissler 2009). To address misfortune and illness, living kin can serve as proxies to take up inappropriate or incomplete actions of past relatives, which through completion diminishes the burdens that they and other surviving family inherit (White 2001). We use the frame of redemption to describe such acts to make amends for, recover, and heal those who came before. They function on multiple levels, including personal, familial, communal, ancestral/spiritual, and for future generations.

The work of redeeming the challenges and misfortunes of the ancestors is relational and reciprocal. Healing inherited issues provides an opportunity for individual and communal healing, which is understood to heal “up” the bloodline (to ancestors) and “down” the bloodline to future generations. The work of healing and pleasing the ancestors is an investment in the well-being of oneself and one’s family across time.^3^ By not doing so, misfortune and difficulty continue to be experienced.

The following interview with a traditional healer about young men elucidates the belief that unresolved ancestral trauma may manifest itself in a variety of personal, familial, and social challenges, and that such trauma must be dealt with to heal:

Gogo Nosi With guys … (ages) 16 to 30. That age group, they just have their own dilemma. … Drinking, smoking, going to prison, crime.

Interviewer What is happening with them?

Gogo Nosi That’s what I want to know actually, because we can’t keep treating the tree without actually finding out, is it the soil? Or what is it? We need to find the root … we can not keep treating the tree when you don’t know what is wrong. … Africans, we carry out our old people’s (ancestors’) baggage. We carry trauma and what my forefather was doing, I will carry that load. And if I can not deal with it the correct way, I will end up doing things I shouldn’t be doing.

Interviewer So you think they are acting out their ancestor’s issues?

Gogo Nosi Exactly.

The belief presented here is that to heal societal ills and personal challenges, it is important to identify and address ancestral misfortunes and traumas. In doing so, people can stop “carrying their load.” Beyond creating more comfortable realities, ancestral healing also involves the possibility of different futures. This quest for redemption is not solely about healing oneself and one’s lineages. There is an impetus to go beyond what came before, to move forward into new states, to grow, and evolve. In this way, ancestors’ lives may serve as a platform that those who come after can stand on, build on and grow. Within this context, illness and moral and social decay are considered collective, relational, and intergenerational, as are the possibilities for healing.

### Blood

Blood—as a physical substance and metaphor—holds importance and meaning in relation to both kinship and HIV. First, it represents the biological bond between living kin and ancestors (Mokgobi 2014), as well as the aforementioned belief that family issues are patterned and passed down through generations. Second, HIV is a virus of bodily fluids—including blood—that can also be transmitted in families, including from parents to children. It is considered to be a virus that both lives in the blood, as well as within kinship relationships and family (Henderson 2011).

#### Case Study: Khwezi.

Below we present the case study of Khwezi^4^ (age 20) to introduce common themes discussed in this article of: (1) parental death; (2) living with HIV; and (3) absence of fathers and patrilineal kin. We present data from Khwezi’s life history narrative interview, clinic file, and in-depth semi-structured interview to provide a window into these themes, as well as to demonstrate how data from multiple sources were triangulated and interpreted.

**Figure 1.**
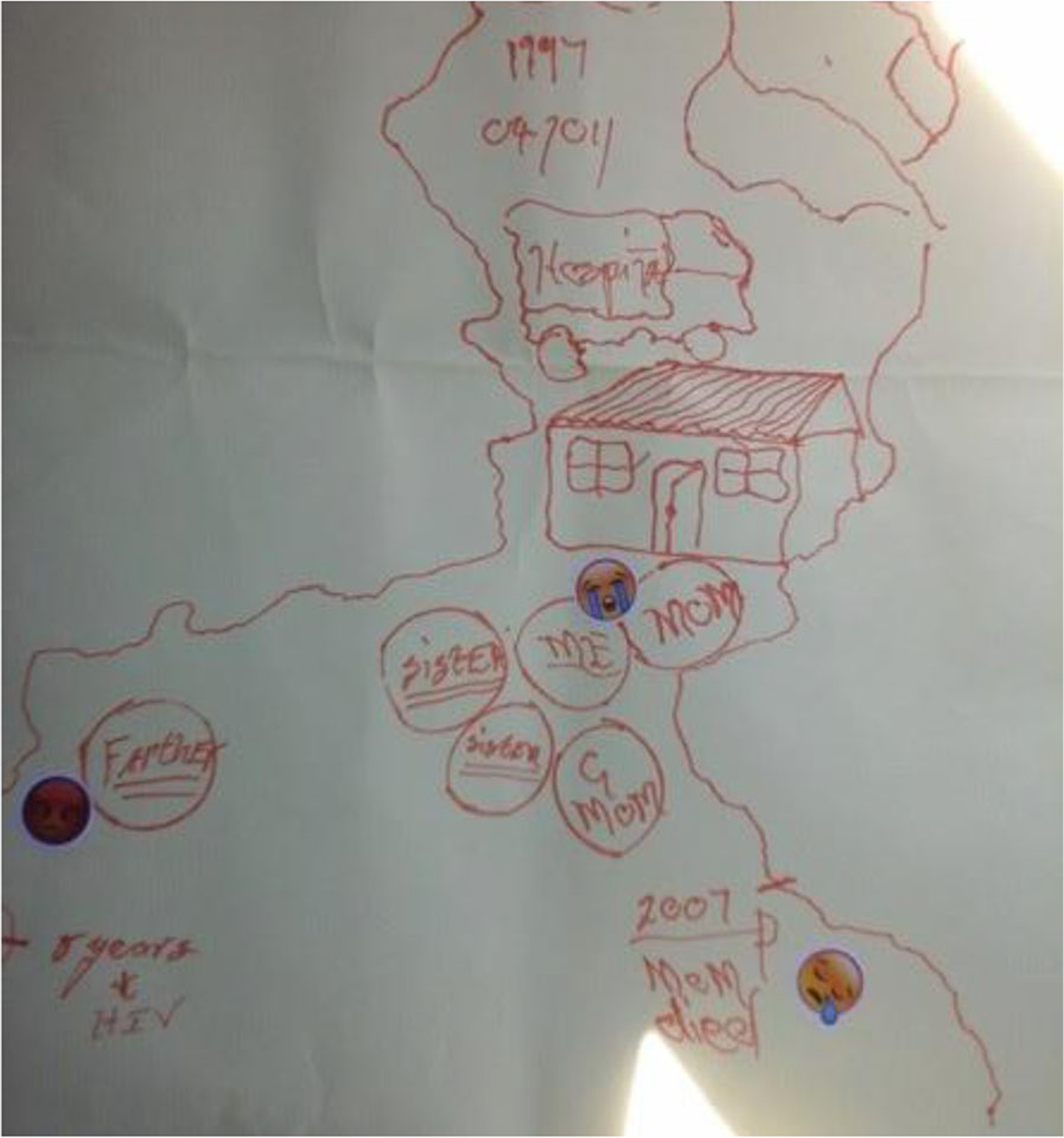
Khwezi’s (20) life history drawing (left). October 6, 2017.

As can be seen in the left image of Figure 1, Khwezi traced objects to represent his physical and emotional proximity to his sisters, mother, and grandmother, living together in his childhood home. He visually demonstrates his closeness with his mother and used crying emojis to demonstrate his sadness over her death. He engaged metaphors from the items that he selected to describe her (e.g., a “diamond”—a clear shiny piece of glass). Visual and verbal context is provided by the emojis and his spoken interview.

Interviewer Let’s talk about your life events, let’s start with an important one. …

Khwezi Firstly, it was the death of my mother, it was like being dumped by your precious thing.

…

Interviewer Why did you put item there (gestures to clear round stone he used to represent his mother)?

Khwezi She was like a diamond to me. … Ever since the day my mother died that day will always be in my mind. … I don’t know the exact date but I know the exact words … (they said that) I had to accept that Luluama is gone and I won’t see her again.

Khwezi’s events were also reflected in other forms of data from different perspectives. His health facility file documents his mother’s death, alongside his caregiver relationships and material situation. His grandmother, depicted as close to him in his drawing, is recorded as his primary caregiver and receiving an old age pension grant. The details of his finding out about his HIV-positive status (his second life event) are also included in the clinic file. His file documents his very low CD4 count (29), third-stage HIV infection, and pulmonary tuberculosis, demonstrating that he was physically sick when he was diagnosed with HIV and providing context to his narrative of this event as a challenging time. His father is also recorded as dead. In his life history narrative, he did not speak about the death of his father, although he spoke about the absence of his patrilineal kin. He visually depicted his father as outside his immediate family unit and he used an angry emoji to represent his feelings. In explaining his anger, he spoke about how not having received support from paternal kin to perform rituals for his paternal ancestors may affect his well-being and success:

Khwezi Not knowing my father at this certain age it affects me because I need things like rituals from my father’s side. I know that I’ve done circumcision with rituals from my mother’s side … at this certain age I have to know my father’s rituals that could affect me. That is why I regard this as a life event.

Interviewer Did you try to reach out to his family …?

Khwezi His family knows that their son has a son … that I’m a man and I have needs concerning my manhood, they even know my family in Phakalani. …They didn’t even show support…

(Khwezi, 20)

In the next section, we present findings from life history narratives with a focus on HIV, parental death, and related financial and geographic precarity. We suggest that these challenges are understood as intergenerational misfortunes that participants aimed to redeem and re-signify through “good” patienthood. Following this, themes in relation to paternal kinship, future ideation and masculine identities are presented, alongside the suggestion that performances of “respectable” adult masculinities serve redemptive functions as participants get older.

## Childhood Redemption through “Good” Patienthood

### Silent Burdens: Parental Death, Precariousness, and HIV

**Figure 2.**
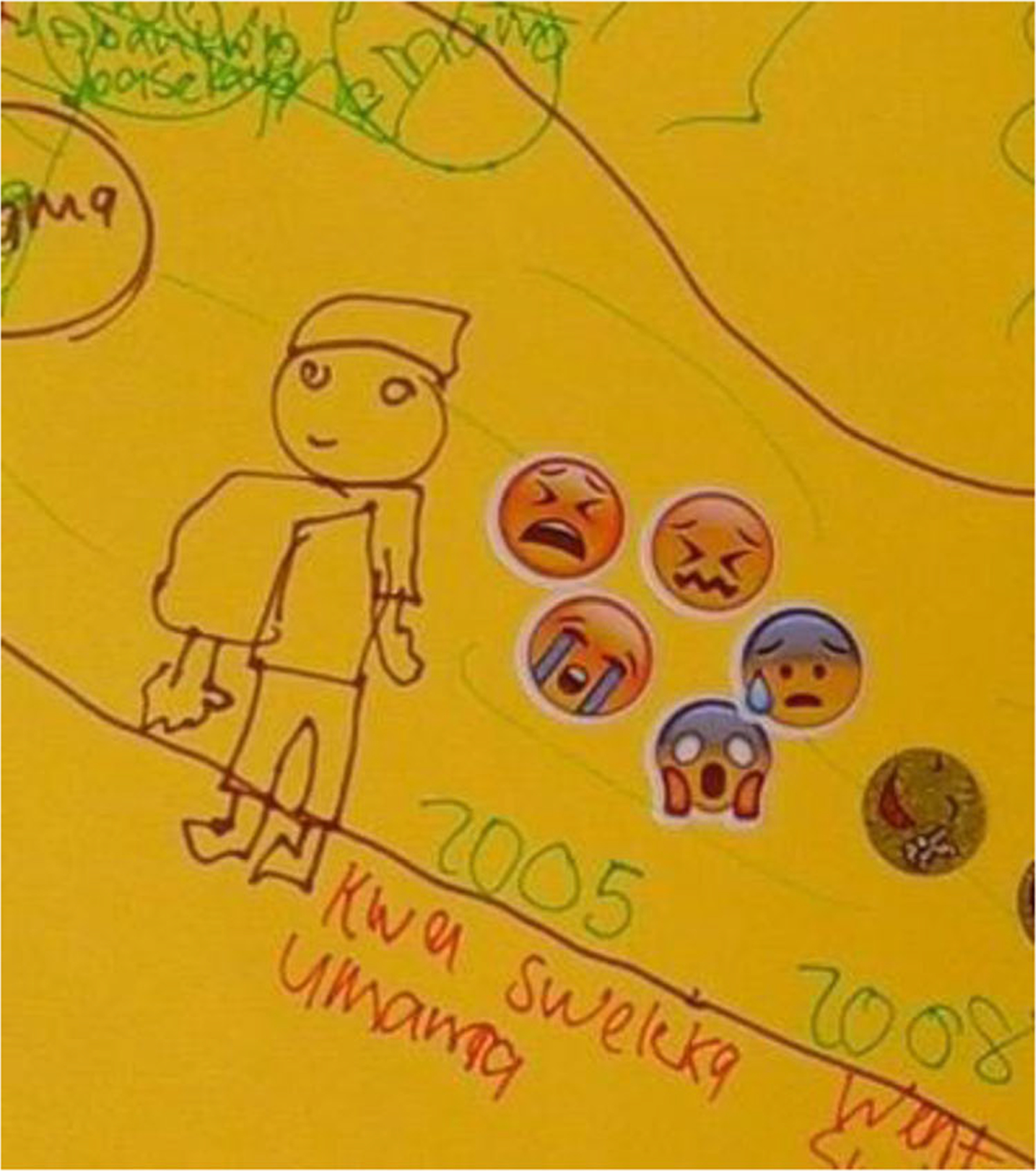
Jeveno* (17) depicts the death of his mother using crying and sad emojis. It says “My mother passed away” (translated).

Death of one or both parents was a strong theme in life history narratives, with 18 participants describing it as their first important life event (see Figure 2). Of the 35 participants in this study, 26 had lost parents, in line with South African trends of AIDS orphanhood (Richter and Morrell 2006).

**Figure 3.**
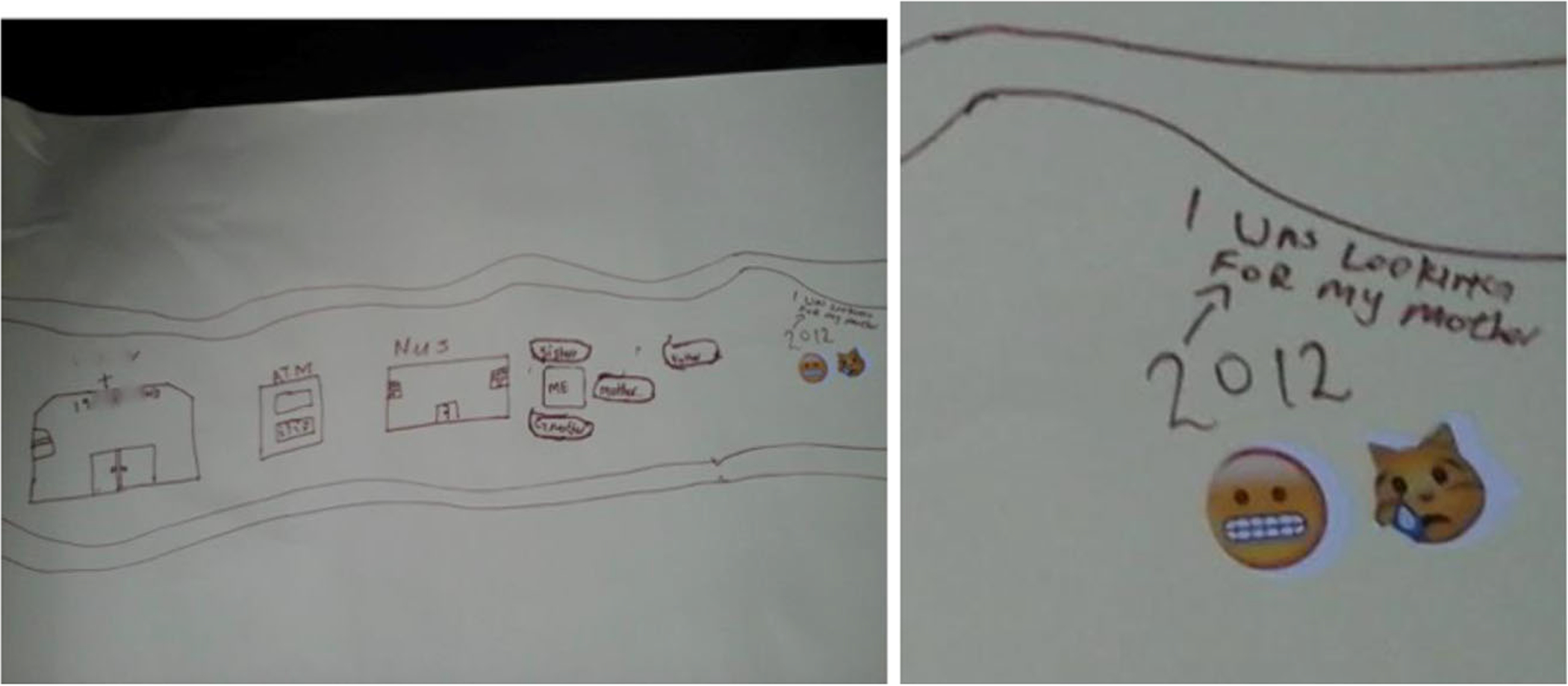
Buja* (19) depicts looking for his mother using a crying and confused emoji.

Narratives of parental death were imbued with pain and tenderness. Many participants described not being explicitly informed about these deaths and being moved without explanation to live with other family members. They described finding out much later, or not being involved in conversations or ceremonies to grieve. This dovetails with the literature on parental AIDS-related deaths in southern Africa. Children are often not told about parental death (see Figure 3) or involved in ceremonies, with the intent of sparing them from emotional trauma and for reasons of pollution, misfortune, and witchcraft (Van der Heijden and Swartz 2010). Children may be seen as too vulnerable to know about parental deaths, and caregivers may feel helpless and unable to manage children’s distress (Van der Heijden and Swartz 2010).

An added element of deaths being AIDS-related made speaking about them even more challenging. Niehaus and colleagues (2001) posit that HIV is stigmatizing because of its relationship to death, and Posel and colleagues (2007) document how AIDS-related deaths are considered a form of “bad” death, an unspeakable sign of moral and social decay. Given that most participants had inherited HIV from their mothers, the death of their parents was related to their own possibility of becoming sick and dying from AIDS-related illness.

Participant narratives revealed confusion and anger over not being told about the death until they were older. In a society with entrenched age-based hierarchy, they felt unable to break this secrecy and did not discuss these topics in their homes. In this way, HIV remained an omnipresent, yet invisiblized reminder of familial misfortunes.

The destabilizing effects of parental deaths were experienced alongside emotional, geographic, and material challenges. Most participants had complex narratives of changing homes and caregivers, and confusing, highly geographically and materially precarious lives. Although children are often raised by grandparents or other family members, in these cases, such high mobility was often related to parental death, financial instability, and family tensions. Shifting caregivers often meant that they were less materially secure, and some described not receiving as much food as other children in their households and fearing not having basic needs met. Beyond affecting grief in complex ways, parental loss also shapes access to social capital, services, and networks to support coping (Cattell 2001). Orphans’ anxieties over social and economic conditions, fear of not having material and emotional needs met, and concerns about getting help with problems have been described elsewhere (Van der Heijden and Swartz 2010).

Learning of their HIV-positive status was another strong theme, discussed unprompted by 32 participants. They described growing up deeply embedded in the biomedical health system, including experiences of severe illness and hospitalization, frequent health facility attendance, interactions with biomedical health workers, and daily pill-taking. Entanglements in this set of biomedical engagements meant that HIV was hyper-present in their lives. However, their HIV-positive status, mode of acquisition, and parental AIDS-related deaths were largely unspoken within homes and families.

### Silent Burdens, Symbolic Misfortunes

Spiritual considerations are often seen to underlie historical and political–economic processes. Traditional African aetiological explanations of illness may incorporate biomedical understandings while drawing on traditional knowledge, culture and spiritual beliefs to look for “ultimate” causes. Niehaus (2009) documents narratives of degeneration of social and ethical standards in South Africa, and HIV/AIDS has been suggested to be seen “but one of the scourges that have befallen Africa over the past century” (Becker and Geissler 2009). Talk of AIDS is often imbedded in narratives of confusion, destruction, and loss, which resonates with broader stories of misfortune (Cohen and Odhiambo 1989). AIDS-related deaths are often considered as a part of “long procession of misfortunes” (Becker and Geissler 2009: 11) rather than a single event. HIV may represent misfortunes of a supernatural nature such as unresolved ancestral issues, witchcraft, violations of cultural taboos, or ancestral displeasure (Shisana et al. 2014; Zungu 2013), and may be understood to be a new form of other long-standing, traditional illnesses (Posel et al. 2007). Alongside the uptake of increasing biomedical HIV-related information over the past decades, traditional beliefs continue to exist in relation to the underlying causes of HIV. For example, Zuma et al. (2018) documents multiple sources of HIV-related belief and healing.

We suggest that the intergenerational silence and stigma around parental death and HIV-positive status documented in this study represent both a hard-to-keep secret, and an implicit collective wound in need of healing. All participants held strong biomedical HIV knowledge, but about half related underlying causes of HIV to something supernatural. Such beliefs were also presented in implicit, indirect ways, using coded and distancing language when discussing family issues, HIV and societal and moral degeneration. Some traditional health practitioners also understood HIV as an intergenerational issue with supernatural origins.

HIV … usually it is about the home. You find out the mother is HIV-positive, the daughter is HIV-positive, then you find out in that household there is a lot of drinking, there is casual sexuality, there is pandemonium. When I look at those things, I find out there is a curse.

[Tamella]

Within this cosmology, illness is relational, occurring in families and symptomatic of an underlying issue that can manifest in many ways, including physical or mental illness, family problems, poverty, employment troubles, social issues, and substance abuse. Many such issues were presented in life history narratives including parental death, financial and geographic precarity, and living with HIV. These may be seen to represent familial misfortune, carried by and passed down from their parents.

Like parental death, participants were often told after a long period of silence about their HIV-positive status. The reasons for these late disclosures are likely similar. Having acquired HIV from their parents, they inherited the same misfortunes, alongside the possibility of an early bad death, from which their families were unable to protect them. Sometimes explicitly spoken, but often implicit, was the awareness of their own mortality that came with living with the inherited chronic condition that their parents died of. Blood as substance and metaphor in relation to kinship and HIV is relevant here. It cements relatedness, and the presence of HIV in the body that “cannot be wished away or denied” (Henderson 2011: 21).

Pervasive silences around HIV and death obscured unresolved intergenerational familial misfortune. Participants kept questions to themselves about their parents’ deaths and their own HIV-positive status. In doing so, they avoided placing blame and defying family authorities by revealing what had been deliberately hidden. Many expressed missing information about their HIV acquisition but were unable to ask for more information from their families. Zube, 17, said: “I was very surprised. … I didn’t ask (how I became infected)” and Layzdu, 18, said: “Why do I have this because I never did anything with anyone?” Others, such as Ringo, 17, explained that his grandmother supports him to take his treatment but never talks about HIV. Mayor, 16, was told of his condition by a health worker, and decided to ask his caregiver: “I once asked her about the pills, and she didn’t answer me. She just cried and called the neighbors.” He never asked again. Health facility files also documented these silences in the form of late disclosures of their HIV-positive status and not being provided with clear information by families.

Given HIV-related stigma, including its relationship to death, immorality, and misfortune, it is not surprising that the HIV-positive status of a child and the AIDS-related death of a parent were met with silence within families. Beyond the suggestion that caregivers need to accept a child’s HIV-positive status to support them, caregivers and family members may feel guilty for not being able to protect the child from their illness and misfortune, as this reflects on the well-being of the family and their failure to ensure healthy progeny. For living biological parents, this might reflect their own HIV-positive status and misfortune that they had passed on to their children. For caregivers of orphaned children, they may have trouble accepting the HIV-positive status or AIDS-related death of a family member of loved one.

### Redemption, “Moral” HIV Acquisition and Health Citizenship

While being born with HIV and losing parents were outside of participants’ control, medicine-taking was within it. A form of “responsibilized” health citizenship, medicine-taking was a way to gain legitimacy and acceptance in the eyes of caregivers and health facility staff and to seek futures different from their parents. The term responsibilized citizen (Barry et al. 1996) was introduced into South Africa as “health citizenship” (Robins 2008). A variation of this idea, “therapeutic citizenship” was developed by Nguyen (2005) in the context of West African NGO-led HIV treatment programs. In a recent literature review on citizenship and ART, Paparini and Rhodes (2016) document multiple contexts of ART provision in which reciprocity for the receipt of ART requires patients to give back through adherence, clinic attendance, and allegiance to its biomedical rationalities. For example, Whyte et al. (2013) documents a social patronage system in Uganda in which people accessing ART are considered in a contractual obligation, with little space for negotiation. Such neoliberal public health discourse suggests that rather than having biological needs met and rights recognized, people are individually responsible for their health and to the state from which they seek inclusion (Paparani and Rhodes 2016).

Participants leveraged notions of therapeutic citizenship through performing pill-taking and health facility attendance. They expressed a sense of duty for the support they received from biomedical health workers and caregivers, and framed pill-taking as an expression of love and gratitude. For participants concerned over not having basic needs met, or who were in shifting or strained caregiver situations, this may be an attempt to ensure survival. Participants also spoke about their obligation to make things right between themselves and health workers, who were central authorities in their lives. This could only be achieved by complying to their demands to adhere to ART and appointments. Health citizenship was also prescribed to the families and caregivers of participants through silence-rupturing interventions of psychosocial support, HIV disclosure, and monitoring pill-taking.

Becoming a good patient through medicine-taking and adhering to clinic appointments can represent the process of becoming a political being. We suggest that these biomedical engagements were also performed for social and spiritual purposes. For adolescents living with HIV, managing their HIV-positive status differently than their parents may function to redeem familial misfortunes. Bernays and colleagues (2017) documented adolescent ART non-adherence being likened to sin, while Vale (2017a) suggests that HIV-positive adolescents are encouraged to take ART to heal the moral degeneration represented in an HIV-positive status and redeem to their lost mothers. Complying with pill-taking can serve the function of redeeming parents who may have disappointed their families or were unable to take medication (Ibid). Participants spoke about medicines-taking as choosing to live, in contrast to parents who had died. By managing their HIV-positive status through pill-taking and health facility attendance, participants can redeem parents by doing something that they did not. In response to the misfortune represented by their HIV-positive status, participants used frames of therapeutic citizenship to put adult authorities at ease, distance themselves from the possibility of their own AIDS-related death, re-signifying their HIV-positive status, and redeeming their parents and lineages.

ART also represents a pathway to (healthy) living and the attainment of collective dreams of the new South Africa. As young citizens, the biomedical health system is both a form of bureaucratic democratic inclusion and a disciplining authority of state control (Vale et al. 2017b). Biomedical products and services take different meanings within sites of home and family, offering different paradoxical and relational forms of citizenship where participants strive to be good and fulfill family responsibilities by taking pills. For younger participants, it was through finding meaning, often in the form of therapeutic citizenship, that they imagined different futures and strove to create new realities. The suggestion here is not that participants had exemplary adherence to ART or health facility appointments, or that they did not take ART for physical benefits. Rather, they strove to be seen to be good patients, regardless of their health practices.

## Redemption through Respectable Masculinity

While participants sought to redeem lost mothers through good health-seeking behaviors, respectable masculinity was a pathway to recuperate fatherhood and become respectable adult men. This section considers aspects of misfortune bound up in issues of identity and masculinity, reporting on complicated, layered, and sometimes conflicting understandings and performances of masculinities in relation to redemption and personhood.

### Fatherhood: Forging Patrilineal Ties and Intergenerational Identity

Here we explore issues of fathers who did not achieve fatherhood in the eyes of participants, either through insufficient presence or provision, or who died, or were unknown. Half the participants had either dead or unknown fathers. Of the others, most did not live with their fathers, and many met them for the first time immediately prior to ulwaluko.

Participants spoke about the absence of fathers and paternal family members with frustration and concern for two interrelated reasons: issues of kinship and identity, bound up in patrilineal family ties, and the material and emotional impacts of these absences. The importance of knowing one’s patrilineal ancestry increased with age, as connecting with patrilineal ancestors may be a prerequisite to fulfilling requirements of respectable adult manhood. Connecting with patrilineages has both preventative and supportive functions. Being known to their ancestors, they can call on them for support, receive protection, and avoid misfortunes that come from ancestral disconnection and sicknesses of identity. The underlying belief here is that through a strong relationship with the ancestors—including through performing ceremonies to connect with them and request their support—longstanding difficulties ranging from material challenges to health and relationship issues can be addressed or avoided. For these reasons, many youth search for their paternal families and surnames (Nduna and Jewkes 2011). This is an example of how unseen elements of personhood may play a role in shaping the kinds of people that boys and men become (Mfecane 2018; Schrock and Schwalbe 2009).

For the participants who had unknown patrilineages, disconnection from paternal ancestry and concerns over accompanying consequences were common. Such absences represented vulnerability and challenges in relation to identity, which may manifest themselves in the form of negative life events.

Two ceremonies were discussed as important to identity. Imbeleko is performed to introduce them to the family, community, and ancestors (Bogopa 2010). Associations between not performing imbeleko and misfortune are common (Ramphele 2002). Ulwaluko is a rite of passage from boyhood to manhood, the ritual of incorporation that will legitimize them as adult men (Mager 1998). It entails circumcision followed by a three- to six-week period of separation from society (Mfecane 2016). If not performed earlier in the child’s life, imbeleko should be performed prior to ulwaluko. All participants had attended or planned to attend ulwaluko.

Forging relationships with paternal families just before ulwaluko was common. Fathers or paternal family members would emerge for support with imbeleko and other prerequisites to ulwaluko. The importance of this introduction to the ancestors is manifold. The initiate should be known by the ancestors before attending ulwaluko, so they can support and protect him during this challenging and potentially dangerous rite of passage. He should also be able to call on them for support and acknowledge them, using their names. As ulwaluko is the gateway into manhood, knowing the ancestors will be important for next phases of life, as successful completion is necessary for getting married, attending and performing ceremonies for the ancestors, and having legitimate children to carry on family names. This aligns with Menkiti (1979) on how rituals of incorporation serve as pathways towards personhood.

Traditional health practitioners also explained that being connected with one’s lineage was important for reasons of protection and well-being, sexual performance, getting married, and having children. Without these connections, men may be impeded, unable to conform to certain norms that legitimize them as men.

**Figure 4.**
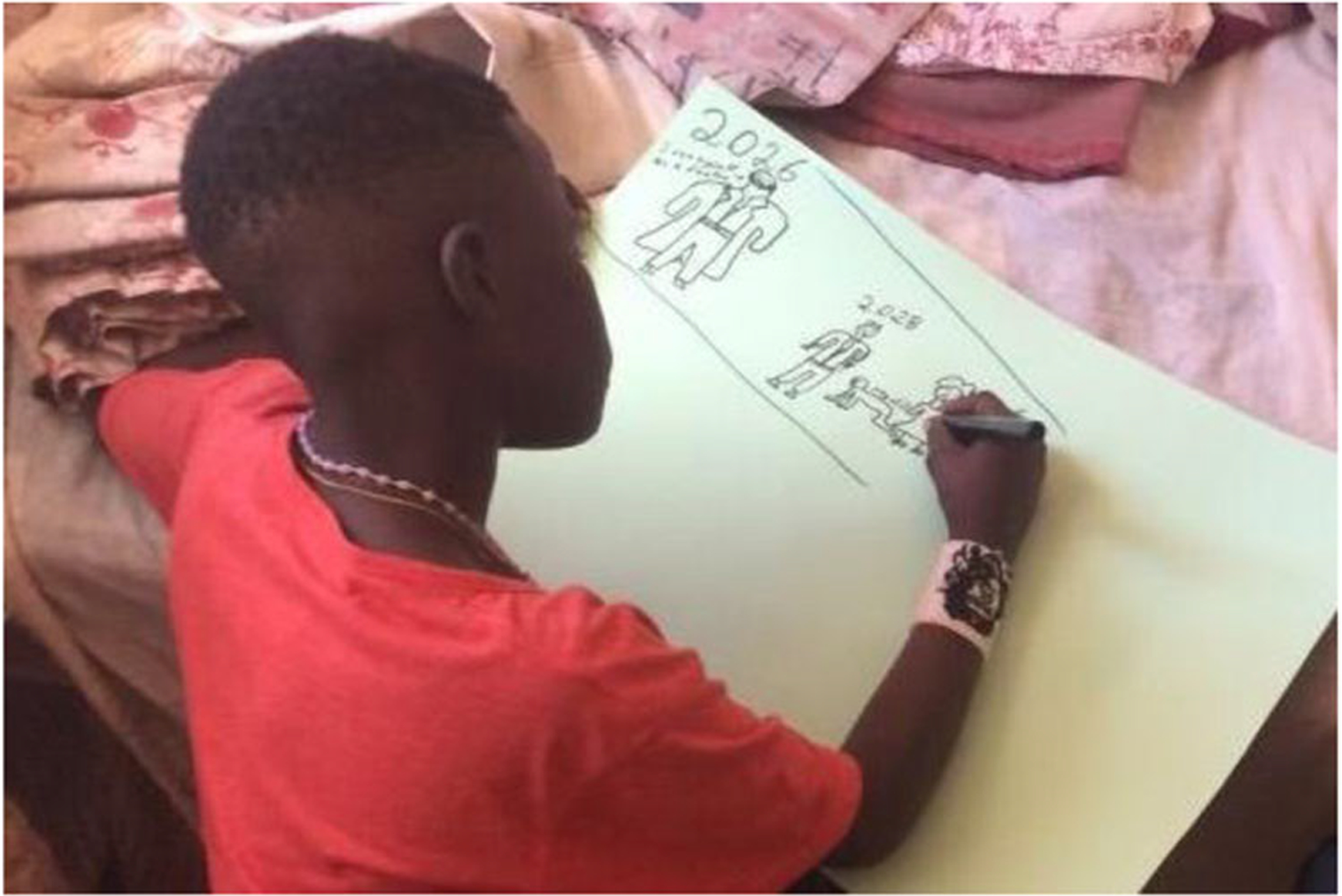
Ulwazi* (13) drawing himself in 2026 as a doctor and a man wearing a suit with his wife and child.

The absence of fathers or paternal family members thus brings up challenges and questions in relation to identity, masculinity, and ultimately the ability to become persons. If they do not know their identities as they get older, their ambitions and abilities as adult men might be thwarted. Issues of absent fathers, aggravated by historical and societal conditions as well as by HIV, represent barriers toward desired futures and adult male personhood. Forging relationships with patrilineal kin was an important foundation for stable and healthy adult male lives (see Figure 4).

### Masculinity, Redemption, and Personhood

Almost all participants wanted children, and spoke about having a female partner, legitimized as a wife, to have children with. This strong presence of norms related to the sanctioning of partnerships via marriage requires substantial financial input for *lobola* (bridewealth). These norms are bound up with frames of respectable masculinities, including financial stability, marriage, social reproduction, and having progeny that will carry on their names.

**Figure 5.**
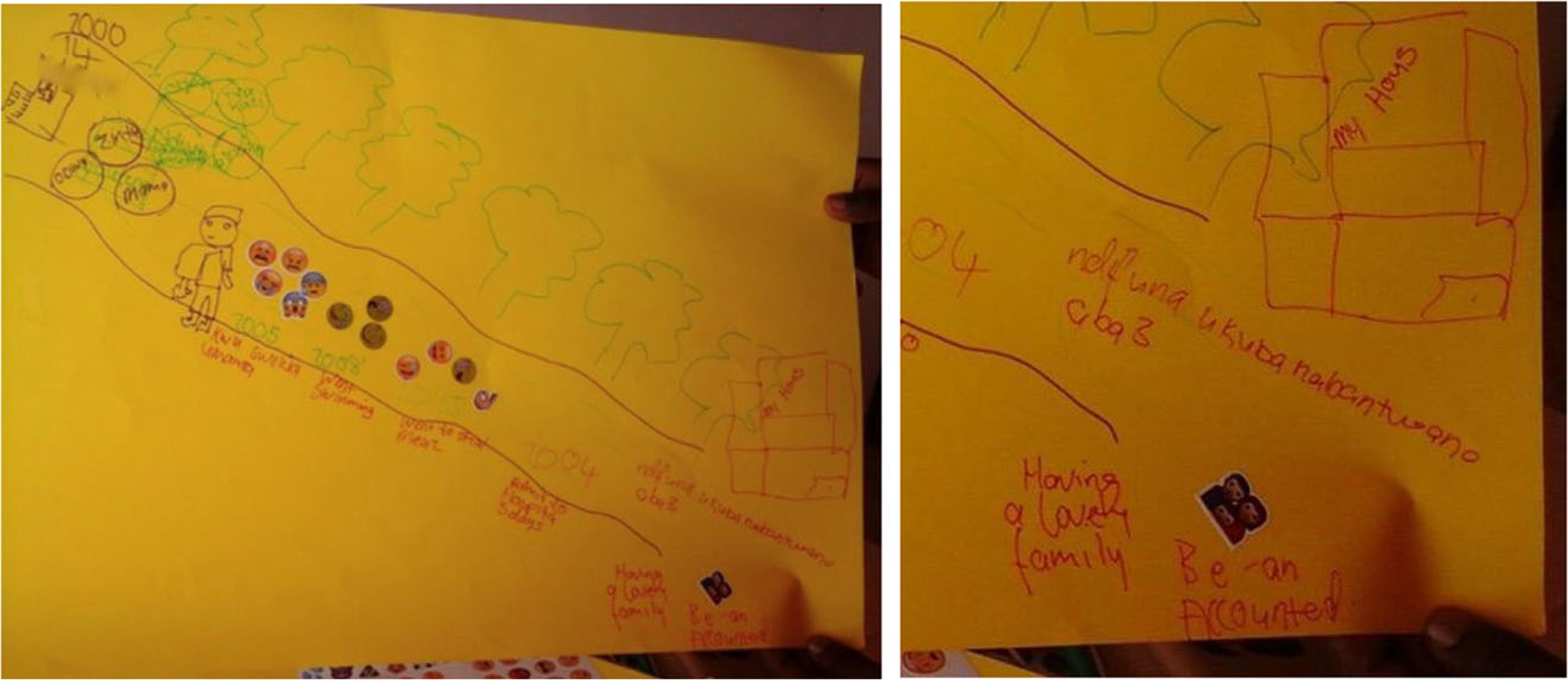
Jeveno* (17) depicts his hopes for the future

The example below has the words “having a lovely family.” accompanied by an emoji of a man, woman, and child huddled together happily. Beside this, he wrote: “be an accounted” (accountant), and above, “I want to have 3 children.” In the top right corner he drew a sizeable concrete home with big green trees. This image demonstrates how participants hope for professional and financial success and families (Figure 5).

Drawing on responsibilized narratives of fatherhood, participants spoke about the timing and the number of children they wanted in relation to finances, demonstrating strong awareness of the costs of children, and desires to act as material providers. Findings align with Mkhwanazi’s (2014) suggestion that young South Africans reproduce and interrupt gendered norms through childbearing. Economic aspirations were tied up in family and gendered provider roles, with breadwinning and “heading” of the family as key markers of masculinity (Richter and Morrell 2006). Participants also spoke about wanting to be present, supportive fathers in contrast to their own experiences as children.

The connection between material provision, physical, and emotional presence is important. Given high unemployment, it is difficult for men to be primary providers and occupy the traditional patriarch role (Bank 2001). Men who cannot pay lobola or damages for impregnation may be seen as men without power (Hunter 2006). Frustration over not being able to meet fatherhood-related social roles such as material provision, can undermine men’s sense of value and success (Hunter 2006). This is especially true for poor men and those unable to live up to expectations for reasons of HIV and illness.

HIV/AIDS has weakened family structures (Richter and Morrell 2006). For AIDS orphans, becoming fathers may represent survival and the ability to carry on their lineages. In a context where living with HIV is considered a barrier to intimate relationships, children, and breadwinning, becoming a father may also symbolize something that is considered outside the realm of HIV-positive masculinities. Where men do not have material resources to begin families within traditional institutions that legitimize them as men, an HIV-positive status may be considered “a mark of their diminishment, a biological manifestation of their social uselessness” (Steinberg 2013: 506).

Engaged, emotionally, and financially present fatherhood may be an avenue toward respectable adult manhood and redeeming failed patriarchs. Although this is not the only pathway, such forms of fatherhood serve the functions of social reproduction, displaying respectable adult masculinity and material security, overcoming the perceived limitations of being an HIV-positive male and redeeming challenging childhoods and patrilineal shortcomings. For most, these key markers of masculinity, including providing for families and having legitimized progeny, were unachieved by their fathers—who were absent, deceased, or living in poverty—and unable to take up the role of a legitimatized patriarch.

A central challenge for participants was to shift their financial realities to survive, support family, and become self-sufficient adult men. They consistently spoke about wanting to find work or pursue further education and described work and financial challenges as the most difficult aspects of their lives. In their desired futures, most wanted to become professionals, build or fix family homes, be financially independent, and provide materially for families.

There is a pronounced contrast between these desires and realities of young men in the Eastern Cape, which sees 36.8% of men ages 15–34 unemployed and lacking education or training (Statistics South Africa 2019). Under apartheid, education in the Eastern Cape was intentionally poorly resourced. Despite significant changes with the advent of democracy, school pass rates and education infrastructure remains poor (Ncanywa 2014). Most participants had dropped out of school or were performing poorly and were years behind. Others were unemployed. The majority lived in female-headed homes where the main income was government grants. Shame, stress, depression, and social identity challenges related to unemployment were evident. Following apartheid, men are increasingly on the peripheries of domestic reproduction, with more women working outside the home and female-centered homes becoming more common (Casale and Posel 2002). Although experiences of poverty and precarity are not unique to boys and men, their sense of responsibility to make money and provide for their families may be gendered.

Without material means to legitimize themselves as men, participants leaned on other pathways to masculinity, and a central theme in how they defined respectable adult masculinity was through actions and behaviors. As Luya (17) said: “There are those men who you don’t need to even ask. You see that he is a man in deed.”

Traits and behaviors central to respectable adult manhood were confidence, independent decision making, taking care of the home, self-knowledge, strength, participation and leadership in families and communities, and acting in a way that earns the respect of others. Behaving in a way that upholds the dignity of others and embodying the traits of being respected and respectable were important. Such behaviors have a communal aspect, and participants emphasized the importance of behaving in exemplary ways that reflect well on their families. Similar to how good health citizenship reflected on adult authorities and those who had passed on, respectable masculinity was both a sign of adult manhood and relational personhood. Young men’s respectability is important to protecting the family’s reputation (Msibi 2019). Since traditional respectable masculine norms such as heading a household and supporting a family are largely unavailable to young men, performances of other masculine norms become more important (Gibbs et al. 2014).

Ulwaluko was an important and contested pathway to manhood. As the central organizing factor in Xhosa masculinity (Mager 1998), some participants spoke about ulwaluko as defining of manhood. Others questioned its role, suggesting that manhood is also grounded in behavior and action. Although participants had varying perspectives about this rite of passage, they all felt strong pressure to complete it as it reflected on them and their families. Although ulwaluko bestows individual benefits, its function is familial and communal.

This section has explored layered and varied understandings and enactments of masculinities, including: (1) aspiring to good fatherhood, defined as being present, loving, and providing materially for children within heterosexual relationships sanctioned through marriage; (2) being financially secure and providing for their families; (3) moral behavior; and (4) successfully completing ulwaluko. It highlights complications and contradictions of masculinities, including the assumption that ulwaluko forms an absolute and irrevocable link to legitimized masculinity. The unprompted assertion by some that ulwaluko was not the sole basis for manhood—and that it is not an absolute marker of masculinity outside of the ways that men behave—demonstrates that many participants understood masculinity to be much more complicated. Similarly, being present and legitimate husbands and fathers and financially successful were not on their own constitutive of masculinity outside of respectable actions.

## Discussion

This thing (illnesses of unknown ancestry), they say it is in the blood. And this HIV, it is in the blood. Blood and blood. (Mamj Ciky Vuza)

The belief that HIV and illnesses of history are intertwined is presented here through the words of Mamj Ciky Vuza, a traditional health practitioner. Just as the knowledge of an HIV-positive status—a virus carried within blood—is deemed within the clinical setting as important for pill-taking and physical health, knowledge of one’s ancestral lineages represents a different importance of understanding of one’s blood. In both cases, being aware of what one carries in one’s blood is considered necessary for well-being. This knowledge underpins actions that can serve therapeutic and redemptive functions. Within this study, there were similarities in how families held secrets in relation to paternal identity, HIV, and parental death. We suggest these were symbolic of familial misfortunes of which participants’ lives were an omnipresent reminder. Through enactments of good HIV-positive patienthood and respectable adult masculinities, the adolescent boys and young men living with perinatally acquired HIV in this study sought to redeem such ancestral misfortunes and achieve personhood.

They aimed to compensate for lost parents and HIV through adhering to norms of good health citizenship as children. As they became older, they searched for identity through knowing their paternal ancestry, and aimed to compensate for failed intergenerational masculinities through performing to the norms of masculinity available to them.

As young Xhosa men from lineages that were impoverished and racialized, they are growing up as “born frees,” the first generation born with democratic freedoms within a former Bantustan homeland. They are also the first cohort of children born with HIV able to grow into adulthood due to access to ART. Coming into adulthood, they feel the pressure to realize livelihoods not available to their forebears.

Although the discourse of the rainbow nation of harmonious race relations and democracy espouses principles of equity and opportunities, unfortunately there is a marked gap between these principles and social and economic realities (Walker 2005). This rainbow nation narrative suggests opportunities are available for everyone to flourish, a stark contrast to participants’ realities and prospects. The promise of these possibilities clashes with the opportunities available to them, creating a myriad of pressures to achieve what is almost impossible in the face of poverty, unemployment, illness, broken patrilineal relationships, and precarious physical and social environments.

This was evident in the pronounced differences between participants’ idealized futures and current realities. Life history narratives focused on precarious financial and geographic circumstances, parental death, illness, and absent fathers. In discussing future ideation, participants shared aspirations drastically different from these experiences and realities. Against the backdrop of neoliberal health and development discourses, participants struggled to be seen as good patients and respectable men while appearing strong and invulnerable. Despite subscribing to norms of masculinity and responsibilized health citizenship, participant behaviors and circumstances often diverged from such narratives in practice. For example, many used substances, did not take their medication or go to the clinic, and were involved in violence and crime—a disjuncture between idealized moral forms of masculinity and patienthood and their actions.

The difficulties of achieving such masculinities, and ultimately, adult manhood, represents a chasm between the aspirational and achievable. Participants never overtly acknowledged the gap between current and idealized realities but expressed desires to close it, sharing their concerns about making money, finding work, and acquiring education. These findings build on a literature that emphasizes the importance of considering multiple intersecting aspects of male identities, and poor and working-class men’s exclusion from the financial system (Dworkin et al. 2015).

Participants also aimed to address this gap through frames of morality, in which they aimed to reconfigure the stigma of HIV, masculinity, and its symbolic misfortunes. They discussed their acceptance of their HIV-positive status in relation to their mode of acquisition, implying that having acquired HIV vertically (and, without choice) was more acceptable than sexual acquisition. Pill-taking and good patienthood represented forms of redemption to their parent’s potentially bad patienthood, or immoral behaviors that had caused them to get HIV (Vale and Thabeng 2016). As participants entered working age, they came up against what it means to be good adult citizens. Despite expressing concerns over their inability to fulfill expectations, they asserted that respectable behavior was a pathway to adult male personhood, and framed themselves as good men by nature of their actions, in contrast to immoral behaviors of other men. Participants engaged frames of morality as redemptive mechanisms—emphasizing that they behaved within communal codes of morality to distance themselves from certain forms of masculinity and HIV-positivity—to situate themselves as moral and respectable persons. The assertion that morality is essential to personhood (Menkiti 2004) is relevant here.

In these ways, participants aimed to liberate themselves from inherited burdens of the past, redeem lost parents and failed patriarchs, and move toward brighter futures in the face of immutable biological and (almost) immutable material realities.

Adolescent boys living with HIV face twin pressures of difficult-to-obtain ideals of good patients and respectable men within lofty but unattainable narratives of freedom in newly democratic South Africa. Their embodied experiences and enacted performances of HIV and masculinity represent the challenges and possibilities of relational personhood for young men living with HIV. Their struggles and aspirations reflect a convergence of idealized neoliberal, hard-to-achieve forms of personhood, including rainbow nation citizenship, and that of responsible HIV-positive patients and young men. With these imagined futures is the realization of possibilities not available to their forefathers for reasons of poverty, HIV, and forced migration under the apartheid labor system.

Participants’ lives and lineages might be seen as a continuum, with familial adversity sitting in the past, and the possibility of realized personhood, progeny and familial well-being in the future. Aspirations for redemption are not solely individualistic, but intricately and irrefutably bound up in their sense of morality, kinship, and personhood. Set against the backdrop of difficult family histories and structural oppression, their health and success would be an achievement for their families and lineages and an offering to their progeny and nation. Blood and blood, the collective hope of transforming a “long procession of misfortunes” into different futures is a heavy burden on their shoulders.

## Footnotes

1. There is large philosophical literature on personhood that encompasses discipline-specific (i.e., justice, bio–ethics) and location-specific theories (i.e., philosophies of personhood in African cosmologies). These span a broad and rich history of philosophy and are thought too substantial to explore within this article. However, concepts presented in relation to personhood are used to interpret and contextualize the data.

2. The term “traditional” as used in this article, refers to traditional amaXhosa cosmologies of health and traditional health practitioners, products, and services. The term “biomedical” is used to refer to public and private health system actors and structures and products and services received through health facilities, and from people formally employed within the health system, including nurses and doctors. These terms are imperfect approximations—engaged as an entry point into health and well-being-related belief systems—with the acknowledgment that such silos do not reflect the ways in which many people conceptualize and practice health-seeking. Although in some ways reifying the binary between traditional and biomedical health systems, this article also suggests that traditional and biomedical health practices are enacted within frames of redemption and personhood, complicating the dominant reporting of traditional and biomedical framings as separate, univocal paradigms.

3. Cosmologies of healing discussed in this article, although documented in the literature, come also from the knowledge of traditional health practitioners including Baba Masechaba (Lindy Dlamini) and her colleagues and lineages with whom the first author trained from 2015 to 2020. Makhosi Thando May and Gogo Nosi Magumasholo and members of the Eastern Cape Traditional Healers’ Association provided insight into traditional cosmologies in the Eastern Cape that undergird this article.

4. All family and place names have been changed to keep participants anonymous.

## Appendix

**Appendix:** Participant Table: Adolescent boys and young men

Pseudonym	Age at first interview	Location	Traditional circumcision status^5^
Unathi*	16 years	BCM, urban	Not circumcised
Ndoda	18 years	BCM, urban	Circumcised
Buja	19 years	BCM, urban	Circumcised during study period
Khwezi*	20 years	BCM, urban	Circumcised
Layzdu	18 years	BCM, urban	Circumcised
Sakhiman	20 years	BCM, urban	Circumcised
Listar	18 years	Amathole, rural	Not circumcised
Ngamla	21 years	Amathole, peri-urban	Circumcised
Machete	22 years	Amathole, peri-urban	Circumcised
Mluthwana	16 years	BCM, urban	Not circumcised
Lullo	22 years	BCM, urban	Circumcised
Mayor	16 years	BCM, urban	Circumcised
Tonxo	22 years	Amathole, rural	Circumcised
Dee	19 years	Amathole, urban	Circumcised
Mr Shade	17 years	BCM, peri-urban	Not circumcised
Ulwazi*	13 years	BCM, peri-urban	Not circumcised
Jeveno	17 years	Amathole, peri-urban	Circumcised during study period
Movite	18 years	BCM, urban	Circumcised
Ta Saider	21 years	Amathole, peri-urban	Circumcised
Nginduyi	16 years	Amathole, peri-urban	Not circumcised
Sne	19 years	Amathole, rural	Unknown. (Planned to go but could not find for a follow-up interview)
Nkweza	18 years	BCM, urban	Not circumcised (rejected at clinic during study period)
Svij	25 years	BCM, urban	Circumcised
C’Vg	19 years	BCM, urban	Circumcised
Sividge	21 years	BCM, urban	Circumcised
Soso	21 years	Amathole, rural	Circumcised
Ndofaya	18 years	BCM, rural	Circumcised
Akhona*	19 years	BCM, urban	Not circumcised (rejected at clinic during study period)
Ringo	17 years	BCM, urban	Circumcised during study period
X-man	22 years	Amathole, peri-urban	Not circumcised (rejected at clinic during study period)
Zube	17 years	BCM, urban	Not circumcised
Luya	17 years	BCM, urban	Not circumcised
Loza	21 years	BCM, peri-urban	Circumcised
Bele	19 years	BCM, urban	Circumcised
Stenza	13 years	Amathole, peri-urban	Not circumcised
